# A risk assessment tool for resumption of research activities during the COVID-19 pandemic for field trials in low resource settings

**DOI:** 10.1186/s12874-021-01232-x

**Published:** 2021-04-12

**Authors:** Suzanne M. Simkovich, Lisa M. Thompson, Maggie L. Clark, Kalpana Balakrishnan, Alejandra Bussalleu, William Checkley, Thomas Clasen, Victor G. Davila-Roman, Anaite Diaz-Artiga, Ephrem Dusabimana, Lisa de las Fuentes, Steven Harvey, Miles A. Kirby, Amy Lovvorn, Eric D. McCollum, Erick E. Mollinedo, Jennifer L. Peel, Ashlinn Quinn, Ghislaine Rosa, Lindsay J. Underhill, Kendra N. Williams, Bonnie N. Young, Joshua Rosenthal, HAPIN Investigators, Vigneswari Aravindalochanan, Kalpana Balakrishnan, Dana Boyd Barr, Vanessa Burrowes, Devan Campbell, Julia Mc Peek Campbell, Eduardo Canuz, Adly Castañaza, Howard Chang, William Checkley, Yunyun Chen, Marilú Chiang, Maggie L. Clark, Thomas Clasen, Rachel Craik, Mary Crocker, Victor Dávila-Román, Lisa de las Fuentes, Oscar De Léon, Anaité Diaz-Artiga, Ephrem Dusabimana, Lisa Elon, Juan Gabriel Espinoza, Irma Sayury Pineda Fuentes, Sarada Garg, Dina Goodman, Savannah Gupton, Meghan Hardison, Stella Hartinger, Steven A. Harvey, Mayari Hengstermann, Phabiola Herrera, Shakir Hossen, Penelope Howards, Lindsay Jaacks, Shirin Jabbarzadeh, Michael A. Johnson, Abigail Jones, Katherine Kearns, Miles Kirby, Jacob Kremer, Margaret Laws, Patricia M. Lenzen, Jiawen Liao, Amy Lovvorn, Fiona Majorin, Eric McCollum, John P. McCracken, Rachel M. Meyers, J. Jaime Miranda, Erick Mollinedo, Lawrence Moulton, Krishnendu Mukhopadhyay, Luke Naeher, Abidan Nambajimana, Florien Ndagijimana, Azhar Nizam, Jean de Dieu Ntivuguruzwa, Aris Papageorghiou, Jennifer Peel, Ricardo Piedrahita, Ajay Pillarisetti, Naveen Puttaswamy, Elisa Puzzolo, Ashlinn Quinn, Sarah Rajkumar, Usha Ramakrishnan, Davis Reardon, Ghislaine Rosa, Joshua Rosenthal, P. Barry Ryan, Zoe Sakas, Sankar Sambandam, Jeremy Sarnat, Suzanne Simkovich, Sheela Sinharoy, Kirk R. Smith, Kyle Steenland, Damien Swearing, Gurusamy Thangavel, Lisa M. Thompson, Ashley K. Toenjes, Lindsay Underhill, Jean Damascene Uwizeyimana, Viviane Valdes, Amit Verma, Lance Waller, Megan Warnock, Kendra Williams, Wenlu Ye, Bonnie N. Young

**Affiliations:** 1 Division of Pulmonary and Critical Care, School of Medicine, Johns Hopkins University, Baltimore, USA; 2 Center for Global Non-Communicable Disease Research and Training, School of Medicine, Johns Hopkins University, Baltimore, USA; 3 MedStar Health Research Institute, Hyattsville, USA; 4 Nell Hodgson Woodruff School of Nursing, Emory University, Atlanta, USA; 5 Department of Environmental and Radiological Health Sciences, Colorado State University, Fort Collins, USA; 6 Department of Environmental Health Engineering, ICMR Center for Advanced Research on Air Quality, Climate and Health, Sri Ramachandra Institute for Higher Education and Research (Deemed University), Chennai, India; 7 A.B. PRISMA, San Miguel, Peru; 8 CLIMA – Latin American Center of Excellence in Climate Change and Health; and Intercultural Citizenship and Indigenous Health Unit, Faculty of Public Health and Administration, Universidad Peruana Cayetano Heredia, Lima, Peru; 9 Gangarosa Department of Environmental Health, Rollins School of Public Health, Emory University, Atlanta, USA; 10 Cardiovascular Division, Department of Medicine, Washington University School of Medicine, St. Louis, USA; 11 Center for Health Studies, Universidad del Valle de Guatemala, Guatemala City, Guatemala; 12 Eagle Research Center, Kigali, Rwanda; 13 Department of International Health, Johns Hopkins Bloomberg School of Public Health, Johns Hopkins University, Baltimore, USA; 14 Department of Global Health and Population, Harvard T.H. Chan School of Public Health, Boston, MA USA; 15 Global Program for Respiratory Sciences, Eudowood Division of Pediatric Respiratory Sciences, Department of Pediatrics, School of Medicine, Johns Hopkins University, Baltimore, USA; 16 Department of Environmental Health Science, College of Public Health, University of Georgia, Athens, USA; 17 Division of International Epidemiology and Population Studies, Fogarty International Center, National Institutes of Health, Bethesda, USA; 18 Faculty of Infectious and Tropical Diseases, London School of Hygiene and Tropical Medicine, London, UK

**Keywords:** Risk assessment, Biosafety, Research, COVID-19, SARS-CoV-2

## Abstract

**Rationale:**

The spread of severe acute respiratory syndrome coronavirus-2 has suspended many non-COVID-19 related research activities. Where restarting research activities is permitted, investigators need to evaluate the risks and benefits of resuming data collection and adapt procedures to minimize risk.

**Objectives:**

In the context of the multicountry Household Air Pollution Intervention (HAPIN) trial conducted in rural, low-resource settings, we developed a framework to assess the risk of each trial activity and to guide protective measures. Our goal is to maximize the integrity of reseach aims while minimizing infection risk based on the latest scientific understanding of the virus.

**Methods:**

We drew on a combination of expert consultations, risk assessment frameworks, institutional guidance and literature to develop our framework. We then systematically graded clinical, behavioral, laboratory and field environmental health research activities in four countries for both adult and child subjects using this framework. National and local government recommendations provided the minimum safety guidelines for our work.

**Results:**

Our framework assesses risk based on staff proximity to the participant, exposure time between staff and participants, and potential viral aerosolization while performing the activity. For each activity, one of four risk levels, from minimal to unacceptable, is assigned and guidance on protective measures is provided. Those activities that can potentially aerosolize the virus are deemed the highest risk.

**Conclusions:**

By applying a systematic, procedure-specific approach to risk assessment for each trial activity, we were able to protect our participants and research team and to uphold our ability to deliver on the research commitments we have made to our staff, participants, local communities, and funders. This framework can be tailored to other research studies conducted in similar settings during the current pandemic, as well as potential future outbreaks with similar transmission dynamics.

The trial is registered with clinicaltrials.gov NCT02944682 on October 26. 2016 .

**Supplementary Information:**

The online version contains supplementary material available at 10.1186/s12874-021-01232-x.

## Background

The spread of severe acute respiratory syndrome coronavirus-2 (SARS-CoV-2) and resulting coronavirus disease 2019 (COVID-19) has led to the temporary suspension of many non-COVID-19 related research activities worldwide. Where feasible, studies are considering remote data collection by telephone or web-based conferencing [1–3]. However, this approach is often not possible when performing anthropometric measurements, specimen collection, or when investigators need to make other direct observations. Even temporary suspension of research activities can potentially cause harm if investigators are evaluating an intervention that is hypothesized to be beneficial. Further, the suspension of data collection could result in loss of study power and potentially introduce bias. Every day, we are gaining a greater understanding of the transmissibility of SARS-CoV-2, and this knowledge increases our ability to safely resume a wide variety of non-COVID-19 related research activities [3, 4]. Where local law or institutional regulations permit activities to restart, investigators need to evaluate the risks and benefits to both research staff and participants of resuming data collection. To safely conduct study activities, researchers need to develop standardized procedures that are based on realistic assessment of these risks, provide guidance on where and when they are manageable, as well as how to minimize the risk with physical distance measures and appropriate personal protective equipment (PPE).

Investigators in the Household Air Pollution Intervention Network (HAPIN) trial initially suspended data collection due to the pandemic in March 2020 and have since restarted collection of behavioural, environmental, biological and clinical measurements during the fifth year of a five-year, multi-country trial [5–8]. HAPIN is a randomized controlled trial in rural areas of Guatemala, India, Peru, and Rwanda that is assessing the health benefits of providing liquefied petroleum gas (LPG) stoves and an 18-month supply of free LPG to 3200 households that otherwise depend on solid biomass fuel (wood, animal dung, or crop residue) for cooking. Measurements of cooking behavior, personal and in-home exposure to air pollution, biological samples and clinical measurements are being collected longitudinally from pregnant women and their newborns in every household, along with an older, non-pregnant adult woman, if she resides in the house [5–8]. Our study involves home visits, as well as visits to health centers and hospitals during the woman’s pregnancy and the first year of the child’s life.

As SARS-CoV-2 spread globally, governments in all four countries implemented public safety restrictions that limited activities to those designated as essential. Essential activities varied across settings and during the initial period of restrictions. Research activities were not considered essential. However, LPG delivery for cooking was considered essential in all four countries. In Guatemala and Rwanda, our research teams were permitted to continue delivering LPG to study households without disruption. In India, the gas companies continued to deliver refill tanks to study participants. In Peru, our team was limited in its ability to deliver gas during the initial weeks of the restrictions, but we were later able to re-establish services with a local gas company for delivery.

With permission to continue delivery of the LPG intervention, we immediately implemented changes in our delivery protocols to minimize SARS-CoV-2 risk. Further, in anticipation of the additional easement of movement restrictions in countries around the world, we reviewed the literature for guidance on strategies researchers have used for assessing the risk of activities during COVID-19 or other pandemics, and found a dearth of available guidance. Perhaps the most relevant existing framework is that proposed by Lumeng and colleagues, which was designed for research focused in clinical settings in the U.S.A. Thus, we developed a risk assessment tool with the guiding principle of ethical research to minimize the potential risks to research staff, participants and rural communities participating in the HAPIN trial research settings. We wanted our risk assessment tool to allow researchers to assess the risk of each study activity utilizing the same general criteria to support management decisions across this large multinational, multi-disciplinary study with both competing and complementary activities. Although our risk assessment tool has been designed within the framework of specific activities of the HAPIN trial, we report here on our approach, which can be applied to other research contexts and questions in similar settings.

## Methods

In developing our risk assessment tool, we drew on a combination of expert consultations, government regulations, national and local expertise, institutional guidance and review of emerging literature. We queried our multi-national panel of investigators and field team leaders from across the trial with expertise in the disciplines of clinical medicine and imaging, nursing, environmental science, epidemiology, behavioral science, community engagement and statistics, along with the trial funders who provide scientific guidance to the HAPIN trial. We sought input from local community leaders, the Ministries of Health, universities and non-governmental organizations regarding appropriate operations and safety concerns. We consulted with the Institutional Review Boards (IRBs) and Data and Safety Monitoring Board (DSMB) presiding over the trial regarding resumption of activities. We drew upon historical occupational health frameworks for infectious disease biosafety and risk assessment and the most recent peer-reviewed and grey literature about infection dynamics. We also considered staff experience. Using all of these inputs, we built a framework to evaluate risk of exposure to SARS-CoV-2 [3]. Our intent was to develop criteria that were clear, simple and actionable for field managers and staff to implement, and to recommend appropriate practices and materials, in accordance with the risk level of each procedure and perceived risk threshold.

While SARS-CoV2 research findings are still emerging, our assessment is based on the consensus that aerosolization and droplet carriage of virus, primarily from coughing, sneezing, singing, crying, talking, are the predominant modes of infection. It is unclear how long the virus remains in the air. Fomites from surface contact may also contribute to transmission, but are likely a smaller risk. Evidence of SARS-CoV-2 presence has been detected in urine, stool, breast milk, semen and blood, but we are not aware of documented transmission through these bodily fluids at the time of this publication. Furthermore, the risk of transmission is greatest in the two days preceding onset of symptoms and continues afterward for at least ten days, and up to twenty days in immunosuppressed patients. Because documented asymptomatic carriage has been widely reported, we assumed that any staff member, collaborator or community participant might be shedding the virus. Small children (especially infants) appear to be infected at the same rate as adults, but have more mild disease and thus may be unknowingly spreading disease. We agreed that viral transmissibility and the true prevalence of COVID-19 are not clearly known in any of our study sites due to limited testing. We also note that recent seroprevalence studies have reported that case burdens are likely underreported. As such, we chose to err on the side of caution and assume moderate to substantial incidence of disease in all our settings. Therefore, risk was defined as large-scale, uncontrolled community transmission. When widespread vaccination has been achieved in our settings and/or when there are other indications of lower prevalence of disease in our sites, we may adjust our risk levels accordingly.

We assessed each HAPIN data collection activity among each group of participants (pregnant woman /new mother, infant, or non-pregnant older adult woman) because the risks may vary with each participant group. Data collection activities were graded and agreed upon by our team of scientists. Local site investigators were asked to report perceived concerns by staff and participants in their communities. Risk factors and definitions were presented to the HAPIN steering committee, which met weekly, for feedback before adoption. Even with adoption by the steering committee, if local community risk factors at the sites did not allow continued trial activities, the activity was stopped until safety could be ensured. Standard Operating Procedures were developed for the resumption of study activities and included guidance on screening staff and participants for Covid-19 symptoms, transporting staff in project vehicles, cleaning equipment and surfaces, conducting home visits and health facility survelliance, and quarantining for suspected exposures to the virus. These documents are reviewed monthly by two assigned investigators on the trial to reflect they reflect the most up to date knowledge of transmission dynamics and local risk.

## Results

Evaluation of risk criteria for each procedure included the age of participant, location, required physical proximity, exposure time, aerosolization potential, and criteria for use of PPE (Table 1). Using these criteria we established a four level schedule that ranged from minimal to unacceptably high risk (Table 2). We then proceeded to assess each research activity according to the criteria outlined in Table 1 and assigned a risk level and appropriate PPE to each of these. We assessed research activities that included LPG fuel delivery, administration of tablet-based surveys (e.g. questionnaires asked of mothers about their children’s health), data downloads from environmental monitors, personal exposure assessment to household air pollution, biological sample collection (e.g. urine, nasal swabs, venous blood) and lab processing of biological samples in the field laboratories, clinical measures (e.g. newborn birth weight, lung ultrasound, blood pressure), observations in homes of pregnant women/new mothers, children, and vascular procedures in adults (Additional file 1).

**Table 1:** Risk Assessment Framework

Risks	Definitions		Example 1- Lung Ultrasound Obtainment	Example 2- Personal Exposure Assessment
Participant	Participant group (e.g. pregnant woman/new mother, child, older adult woman)		Child	Older Adult Woman, Pregnant Woman
Location	Place where sample is collected or procedure performed		Healthcare facility	Home (indoor or outdoor)
Proximity to the participant	*Close:* The procedure requires the field worker and participant to be closer than 2 m (6 ft) of one another. If a procedure produces aerosolization, then it is automatically considered close contact. *Socially distant:* The procedure to be performed allows the field worker and participant to maintain a distance of > 2 m (6 ft) apart from one another.		Close	Close
Exposure time	*Short*: The procedure can be performed without the staff and participant in close proximity for > 15 min. *Prolonged:* The procedure requires the field worker and participant to be in close proximity for > 15 min.		Prolonged	Setup: short to prolonged Take-down: short
Aerosolization Potential	*None:* The procedure is unlikely to produce any aerosolized particles. *Yes:* The procedure may produce aerosolized particles.		Yes	None
Personal Protective Equipment (PPE) Needs	Criteria to determine PPE	PPE Needs	N95 or equivalent respirator + eye protection + gown + gloves	Paper facemask + eye protection + gloves
	Participant and staff are not in close contact at anytime. No aerosolizing procedures. No processing of biologic samples.	Paper or cloth facemask.		
	Participant and staff may be in close contact but only for a short period of time. Biologic materials may be processed in the lab. No aerosolizing procedures performed.	Paper facemask (preferably procedural quality) + eye protection + gloves (if the procedure requires touching the participant and/or a clinical specimen is collected.		
	Participant and staff may be in close contact for a prolonged period of time and/or an aerosolizing procedure is occuring	N95 or equivalent respirator + eye protection + gown + gloves.		
	Participant and staff may be in close contact for a prolonged period of time and/or an aerosolizing procedure is occuring in a manner that staff and participants can not be safely protected.	Procedure will not be performed.

**Table 2:** Semi-quantitative risk schedule

Scale	Descriptor	Definition	Example (see Additional file 1 for full descriptions)
Level 1	Minimal Risk	Participant and staff are not in close contact indoors at anytime. No aerosolizing procedures. No processing of biologic samples.	Data collection by phone, in-person survey administration outdoors, LPG fuel delivery
Level 2	Moderate Risk	Participant and staff may be in close contact but only for a short period of time. Biologic materials may be processed in the lab. No aerosolizing procedures performed.	Brachial artery reactivity testing, carotid artery reactivity testing, blood pressure measurement, fetal ultrasound, personal exposure assessment in adults, blood collection in adults, urine collection in adults
Level 3	High Risk	Participant and staff may be in close contact for a prolonged period of time or an aerosolizing procedure is occuring (e.g. child crying during length measurement)	Anthropometry, collection of blood in children, screening children for pneumonia, lung ultrasound, buccal scrape, nasal brush
Level 4	Unacceptable Risk for Research	Participant and staff may be in close contact with patient samples for a prolonged period of time and an aerosolizing procedure is occuring in a manner that staff and participants can not be safely protected.	No HAPIN procedures were deemed Level 4; however, the following procedures would be deemed Level 4 in our framework: bronchoscopy, induced sputum, cardiopulmonary resuscitation (CPR)

Protective measures available in our settings were: a) where feasible, data collection was completed by telephone; b) where possible, face-to-face activities were conducted outside; c) when inside homes, clinics or offices, staff and participants minimized the number of people in the room; d) rigourous hygiene for staff, materials, equipment and surfaces were employed at all times; e) appropriate PPE was used based on the context and activity; f) under very high risk conditions, the visit or the procedure was suspended.

Using this assessment and taking necessary measures for protection, almost all of our research activities were deemed to pose potentially manageable risks. Biological sample collection spanned a range of assigned risk due to differences in participant-staff interaction. The activities with the highest level of risk were those that potentially aerosolize the virus during the procedure. For example, urine collection requires minimal contact (i.e., field workers instruct the participant to collect and store the urine sample until it can be retreived) resulting in low risk to both the participant and the study staff. However, dried blood spot collection from capillary blood draws from infants (who are unable to wear a mask and often cry during the procedure) could feasibly put field workers at high risk (examples of two procedures are provided in Table 1; all procedures are described in the Additional file 1). To illustrate Level 4 activities, we identified several activities that were not part of our protocol, but that could pose unmanageable risks (e.g. bronchoscopy, sputum inducting procedures, cardiopulmonary resusitation) for routine research in the pandemic context (Table 2).

## Discussion

Our risk assessment framework uses a four-level risk schedule that is flexible, allowing adjustment for changes to risk measures and definitions as new evidence emerges about virus transmission. The approach and risk assessment tool we present here can be adapted by other investigators who are assessing and managing the risk posed in their own research during the coronavirus pandemic. However, prior to the deployment of risk assessment tools such as ours, researchers, in association with community members, IRBs, DSMBs, and grant funders need to evaluate the importance of any activity related to the primary aims of a trial weighed against the associated risk of performing the research activity. Obviously, local health regulations related to mobility, home or clinic visits by researchers supersede any of these judgments.

The framework offers a way to systematically evaluate diverse research activities involving different disciplines using the same basic criteria and a scoring system to compare associated risk for a given procedure. It also provides clear guidance for field teams on the appropriate PPE and practices in the context of limited resource environments, and thus appropriately utilizes limited PPE where it may be scarce and expensive. Despite these strengths, there are limitations. Our framework does not make recommendations on whether or not to continue an activity – e.g. through an explict cost-benefit algorithm. Decisions on what research should be continued in the presence of risk also require a careful assessment of benefits. We chose to make the risk-benefit calculation and decisions regarding which activities to suspend an independent process from assessment of risk. In our context, an efficacy trial, we are in equipoise regarding the potential benefits of the intervention to participants. Thus, analysis of benefits can only be honestly assessed in terms of the potential benefit of a given activity to the integrity of the trial, not to trial participants as maybe the case for other kinds of clinical research. Among the criteria we used to examine potential benefits of risk in the HAPIN trial were whether or not the aim of any given procedure supports a primary, secondary or tertiary (exploratory) outcome of the trial protocol. This evaluation is made by the HAPIN Steering Committee.

Furthermore, we do not factor in specific local regulations into the matrix *in an* a priori *fashion,* and thus leave it to local investigators and study teams to adjust for these [9]. Because of this, our framework specifications may need to be adjusted to meet local institutional or government regulations regarding PPE or other safety practices. Finally, our framework is limited by the current evidence regarding transmission risk and should be reevaluated and updated as our understanding grows. Such updates will require evaluation by scientists who are up to date on the current literature and recommendations regarding transmission and appropriate PPE, and must be sensitive to changes in local practices. While the recently discovered variants of the SARS-COV-2 suggests higher transmission risk, we do not have experimental or observation evidence at this time that our framework should be significantly changed [10]. However, should evidence emerge that for example, cloth masks are less protective or residual survival of the virus on surfaces is greater, we would need to make changes to our protocols [11].

Of note, the most relevant existing framework we are aware of for resumption of research in the COVID-19 pandemic context is that published by Lumeng and colleagues designed for U.S. clinical research. Our framework was developed independently and for a different context, but their basic approach is similar to ours in that it provides for a high level set of principles, a tiered framework, and risk evaluation that includes factors such as duration and distance of contact between researchers and patients. Our framework adds a great detail to risk evaluation in more complex and varied environments, and outlines how these can apply to specific and diverse research tasks (See Additional file 1: Tables 1–5). Our framework also differs from Lumeng et al’s in that we have not included an explict benefit analysis, as described in the preceding paragraphs.

This risk analysis takes place in the dynamic context of a global pandemic. We plan to reassess each activity using our tool at least monthly as more information about SARS-CoV-2 transmission and the local epidemics becomes available. While the pandemic has been disruptive to our research, we believe there may also be some benefits from the shift in some data collection methods. For example, collecting data via telephone instead of visiting in-person increases time use efficiency for staff and decreases the burden of household visits on participants. Costs are lower, with less fuel used to travel via truck or motorbike to distant participant homes. On the other hand, telephone surveys may introduce uncertainty, if questions are complex in nature and may lead to poor response rates or lower quality data [12]. We acknowledge that we have been able to resume study activities in some of our research sites, and attribute this to building relationships with participants over the past several years and the commitment of our local teams and collaborating institutions.

For researchers now facing the need to resume activities that may lead to risk of exposure to staff and participants, we offer the following advice. First, evaluate any scientific developments about risk of infection or severity of disease that might change the calculus fundamentally. Second, convene representatives of your research teams, IRB members, relevant patient groups, stakeholders, and infectious disease experts to evaluate research activities against our framework and risk schedule, and then adapt as necessary with broad input. Third, while the field, clinical and laboratory activities we presented (Tables 1-2, Additional file 1) may be similar in scope to other research activities, we have obviously not presented all of the potential scenarios. Research activities should be adapted to fit individual research needs, reviewed repeatedly by stakeholder groups until consensus is reached, and operationalized using Standard Operating Procedures for each activity stream.

Beyond the risk assessment tool we have outlined above, we offer the following brief description of how we deployed these rules for field teams that may have similar needs. At the beginning of the pandemic, we temporarily suspended all activities except for LPG fuel delivery until risk of the measurements could be assessed and procedures put into place to ensure safety. We continued collecting data by telephone when possible. When in-person contact was permitted by local authorities and local institutional IRBs, we used our framework to guide the appropriate protocols. If designated PPE was not available or could not be used properly at any time, we postponed the activity. Similarly, our rules required goggles or face shields for certain procedures, but participants (especially children) may find these terrifying, especially when combined with masks and gloves. In these situations, it may not be possible to complete the work as planned, and local staff had the autonomy and responsibility to decide whether any activity should proceed.

Finally, our guidance is based on expert opinion and has not been empirically verified at this time. Importantly, our framework does not substitute for the need for coordination and approval of IRBs when protocols are modified.

## Conclusion

We are optimistic that by applying this systematic, procedure-specific approach to risk assessment for each research activity, we will minimize the disruption in our trial due to the pandemic and support the completion of our primary outcomes. Our framework can be applied to other field trials in low-resource settings to guide investigators in assessing the risk of each trial activity and implementing appropriate safety measures, where the level of risk is acceptable. While no activity in the current context is completely without risk of infection, utilizing a systematic approach is the optimal way to safeguard research activities, protect research staff and participants, and comply with the ethical obligations to those that have agreed to participate in trials, along with the communities and funders that have supported these efforts.

## Supplementary Information

**Additional file 1.**

## Data Availability

Data sharing is not applicable to this article as no datasets were generated or analyzed during the current study.

## Abbreviations

SARS-CoV-2: Severe acute respiratory syndrome coronavirus-2
COVID-19: Coronavirus disease 2019
PPE: Personal protective equipment
HAPIN: Household Air Pollution Intervention Network
IRBs: Institutional Review Boards
DSMB: Data and Safety Monitoring Board
