# Supplementary material for: A risk assessment tool for resumption of research activities during the COVID-19 pandemic for field trials in low resource settings
Source: BMC Med Res Methodol. 2021 Apr 12;21:68. doi: 10.1186/s12874-021-01232-x (PMC8040756; doi:10.1186/s12874-021-01232-x)
Supplement: Supplementary file 1 — Additional file 1. [file 12874_2021_1232_MOESM1_ESM.docx]

Supplement

**Each table displays a case-based analysis using the presented framework for aach activity in HAPIN**

| Appendix Table 1:. Materials Delivery, Surveys, Data-downloads | | | | | |
| --- | --- | --- | --- | --- | --- |
|  | Fuel Delivery | Survey Administration | | Stove Use Monitoring (SUMS) | |
| Risks | LPG Tank swap | In-person | Over the phone | Indoor SUMS Download | Outdoor SUMS Download |
| Participant | *Pregnant woman, older woman* | *Pregnant woman, adult woman* | *Pregnant woman, Older adult woman* | NA | NA |
| Location of collection | Home | Home | Office | Home (indoor) | Home (outdoor) |
| Proximity to the participant | Socially distant | Socially distant | Socially distant | Socially distant | Socially distant |
| Exposure time | Short | Short | None | Setup: none  Take-down: none | Setup: none  Take-down: none |
| Aerosolization | None | No | No | None | None |
| PPE Needs | Paper or cloth facemask | Paper or cloth facemask | Paper or cloth facemask | Setup & take-down: Paper or cloth facemask | Setup & take-down: Paper or cloth facemask |
| Overall Risk Score | 1 | 1 | 1 | 1 | 1 |

Supplement Table 1 describes the risk assessment for the delivery of materials which includes the delivering new LPG fuel tanks and removing the empty tanks from homes, administering surveys (i.e. quality of life, CREDI), and downloading data from stove use monitoring devices that are placed on stoves to monitor cooking practies (Clausen et al 2020).

| Supplement Table 2:. Exposure Assessment in homes | | | | | |
| --- | --- | --- | --- | --- | --- |
| Risks | Personal Exposure | Personal Exposure | Indoor Area Sampling | Outdoor Area Sampling | Ambient Sampling |
| Participant | Older Adult Woman, Pregnant Woman | Child | NA | NA | NA |
| Location of collection | Home (indoor or outdoor) | Home (indoor or outdoor) | Home (indoor) | Home (outdoor) | Health Facility (setup outdoors but requires entering indoor areas) |
| Proximity to the participant | Close | Close | Socially distant | Socially distant | Socially distant |
| Exposure time | Setup: short to prolonged  Take-down: short | Setup: short  Take-down: short | Setup: none  Take-down: none | Setup: none  Take-down: none | Setup: none  Take-down: none |
| Aerosolization | None | None | None | None | None |
| PPE Needs | Setup & take-down: Paper/ cloth facemask/ + eye protection + gloves | Setup & take-down:  Paper/ cloth facemask + eye protection | Setup & take-down: Paper or cloth facemask | Setup & take-down: Paper or cloth facemask | Setup & take-down: Paper or cloth facemask |
| Overall Risk Score | 2 | 2 | 1 | 1 | 1 |
| Supplement Table 2a. Exposure Assessment in Lab | | | | | |
| Risks | Data Download | PM Filter handling | PM Filter weighing | BC Optical Assesments |  |
| Aerosolization | None | None | None | None |  |
| PPE Needs | Paper facemask | Paper facemask | Paper facemask | Paper facemask |  |
| Overall Risk Score | 1 | 1 | 1 | 1 |  |
| *All lab procedures are performed by the staff member without physical contact with a participant or other staff member. | | | | | |

Supplement Table 2 describes the risk assesment for the collection of exposure samples in homes. Appendix Table 2a describes the risk assessment for the processing of the samples (Clausen et al 2020 and Johnson et al 2020).

| Appendix Table 3. Biomarkers - Sample Collection | | | | | | | | |
| --- | --- | --- | --- | --- | --- | --- | --- | --- |
| Risks | Urine – main study | Whole Blood (finger stick dried blood spots)- main study | Whole Blood (finger stick dried blood spots)- main study | Whole Blood (venous blood draw)- *NCI substudy* | Buccal cells (buccal scrape)- *NCI substudy* | Buccal cells (oral rinse)- *NCI substudy* | Nasal cells (nasal brush)- *NCI substudy* | Stool (diaper)– *ancillary study* |
| Participant | *Child* | *Adult* | *Child* | *Adult* | *Adult* | *Adult* | *Adult* | *Child* |
| Location of collection | Home | Home | Home | Home | Home | Home | Home | Home |
| Proximity to the participant | Socially distant | Close | Close | Close | Close | Socially distant | Close | Socially distant |
| Exposure time | None | Short | Prolonged | Short | Prolonged | None | Prolonged | None |
| Aerosolization | None | None | Yes | None | Yes | None | Yes | None |
| PPE Needs | Paper facemask + eye protection + gloves | Paper facemask + eye protection + gloves | N95 + eye protection + gloves + gowns | Paper facemask + eye protection + gloves | N95 facemask + eye protection+ gloves + gowns | ‘  Paper facemask + eye protection + gloves | N95 facemask + eye protection+ gloves + gowns | Paper facemask + eye protection + gloves |
| Overall Risk Score | 2 | 2 | 3 | 2 | 3 | 2 | 3 | 2 |
| Appendix Table 3a. Biomarkers - Lab Processing | | | | | | | | |
| Risks | Urine – main study | Whole Blood (finger stick dried blood spots)- main study | Whole Blood (finger stick dried blood spots)- main study | Whole Blood (venous blood draw)- NCI substudy | Buccal cells (buccal scrape)- NCI substudy | Buccal cells (oral rinse)- NCI substudy | Nasal cells (nasal brush)- NCI substudy | Stool (diaper)– ancillary study |
| Aerosolization | none | none | none | none | none | none | none | none |
| PPE Needs | Paper facemask + eye protection + gloves | Paper facemask + eye protection + gloves | Paper facemask + eye protection + gloves | Paper facemask + eye protection + gloves | Paper facemask + eye protection + gloves | Paper facemask + eye protection + gloves | Paper facemask + eye protection + gloves | Paper facemask + eye protection + gloves |
| Overall Risk Score | 2 | 2 | 2 | 2 | 2 | 2 | 2 | 2 |
| *All lab procedures are performed by the staff member without physical contact with a participant or other staff member. | | | | | | | | |

Supplement Table 3 describes the risk assessment for the collection of biomarkers collected from participants.

Supplement Table 3a describes the risk assessment for the lab processing of biomarkers collected from participants (Barr et all 2020 and Clausen et al 2020).

| AppendixTable 4 Fetal Growth, Anthropometry and Child Pneumonia Assessment | | | | | |
| --- | --- | --- | --- | --- | --- |
| Risks | Fetal Ultrasound | Anthropometry- Length | Anthropometry- Birth Weight | Hospital visits to screen for pneumonia | Pneumonia home visits |
| Participant | Pregnant woman | Child | Child | Child | Child |
| Location of Procedure | Health center | Home | Health facility (at birth); In home for other visits | Hospital | Home |
| Proximity to the participant* | Close | Close | Close | Close | Vitals performed: close  No vitals performed: socially distant |
| Exposure time | Prolonged | Short | Short | Prolonged | Vitals performed: prolonged  No vitals performed: none |
| Aerosolization | None | Yes- child crying | Yes – child crying | Yes | Vitals performed: Yes, if child cries  No vitals performed: none |
| PPE Needs | Paper facemask + eye protection + gloves | N95 facemask or equivalent respirator + eye protection+ gloves + gowns | N95 facemask or equivalent respirator+ eye protection+ gloves + gowns | N95 facemask or equivalent respirator + eye protection + gloves + gowns | Vitals performed: N95 facemask or equivalent respirator + eye protection+ gloves +/- gowns  No vitals performed: Paper/ cloth facemask |
| Overall Risk | 2 | 3 | 3 | 3 | Vitals performed-3/ No vitals performed -1 |

Supplement Table 4 describes the risk assessment for activities related to the fetus of the pregnant woman and the infant once the child is born (Clausen et al 2020, Simkovich et al 2020).

| Appendix Table 5: Vascular and Imaging Procedures | | | | |
| --- | --- | --- | --- | --- |
| Risks | Bronchial Artery Reactivity Testing (BART) | Carotid Intimal Media Thickness (CIMT) Measurement | Lung Ultrasound | Blood Pressure Measurement |
| Participant | Older adult woman | Older adult woman | Child | Older adult woman |
| Location | Home, health facility, community center | Home, health facility, community center | Health facility | Home, health facility, community center |
| Proximity to the participant | Close | Close | Close | Close |
| Exposure time | Prolonged | Prolonged | Prolonged | Short |
| Aerosolization | None | None | Yes | None |
| PPE Needs | Paper facemask + eye protection + gloves | Paper facemask + eye protection + gloves | N95 facemask or equivalent respirator + eye protection + gloves + gowns | Paper facemask + eye protection + gloves |
| Overall Risk | 2 | 2 | 3 | 2 |

Supplement Table 5 describes clinical activities and measurements during the HAPIN trial (Clausen et al 2020, Simkovich et al 2020).

Additional Safety Precautions Implementing during the HAPIN trial:

During home visits, we provide adult participants and family members with paper or cloth masks to wear. Study staff maintain at least a two-meter distance between themselves and participants when collecting survey data, and only approach participants for hands-on activities such as blood pressure measurement, certain biological samples, or placing personal air pollution monitoring equipment. We attempt to perform as many procedures as possible outdoors. Participants are screened prior to arrival to the home for symptoms of COVID-19 and staff are screened daily and asked to stay home if there is any concern for COVID-19. When this has occurred, we have followed local guidance on when the quarantine period is complete. Staff regularly wash their hands with soap and water, and bring containers of water to house visits if water is not available, antibacterial hand gel is also used before and after each collection and house visit.
